# Studies on the Non-Protein Thiols of a Human Prostatic Cancer Cell Line: Glutathione Content 

**DOI:** 10.3390/cancers2021092

**Published:** 2010-06-02

**Authors:** Michael Gronow

**Affiliations:** CCRF-Mitchell Laboratory, Cambridge Research Laboratories, 181A Huntingdon Rd., Cambridge CB3 ODL, UK; E-Mail: michael@gronow-cambridge.co.uk or cam.cancer@btconnect.com

**Keywords:** human prostate tumor cells, non-protein thiols, Ellman reagent labeling, analysis, glutathione content

## Abstract

The low molecular weight thiol (-SH) content of a human prostate carcinoma cell line (LNCap), important to the cellular resistance to drugs and irradiation, was investigated using three forms of thiol assay each utilizing different chemistries. The composition of the mixture was examined by derivatization of the thiols with a three-fold excess of the Ellman reagent to give mixed aromatic disulfides. The components were isolated by chromatography on C_18_ reverse phase silica gel followed by DE52 anion exchange separation, and then analyzed by capillary electrophoresis against prepared standards. The glutathione adduct (GSSE) and an unknown disulfide (RSSE) were the major components isolated on DE52 together with two minor ones. However, from the absorbance at 325 nm, it was found that the GSSE isolated (1.5 ± 0.2 femtomoles/cell) could only account for 28.5 ± 4.3% of the total ASF thiols. It appeared that the bulk of the thiol material had not formed a stable mixed disulfide with Ellman’s reagent, and this was confirmed by ^35^S labeling of the cells. A large proportion of the ^35^S labeled components, obtained after reaction of the ASF thiols with the Ellman reagent, did not form mixed aromatic disulfides and could therefore not be identified by this labeling method.

## 1. Introduction

Thiol compounds occupy a pivotal role in cellular metabolism. Much research has been published stressing the importance of thiols in various aspects of cell metabolism [1], particularly with regard to their essential function in the maintenance of cellular redox balance and their role in controlling oxidative stress, gene expression [2] and redox signaling [3]. Furthermore, they have long been known to play a vital role in cellular sensitivity to radiation and chemotherapeutic drugs [4].

In view of this diversity of thiol metabolism it is perhaps a surprising finding that, despite the key role of these compounds in cellular metabolism, e.g., such as coenzyme A, there are few species of low molecular weight thiols to be found in the cellular acid soluble fraction left after the removal of protein and nucleic acids. The current state of the art in the analysis of thiols has recently been reviewed by a number of authors [5], but it is widely believed that the ubiquitous tripeptide glutathione (GSH) is the most abundant intracellular thiol, present in millimolar concentrations. Some investigators estimate that GSH constitutes greater than 90% of the non-protein fraction, but there have been reports that this may not be the case in tumors [6]. Earlier work at the author’s laboratories has confirmed that this may be true but the difficulties involved in thiol analysis, such as ease of oxidation, have hampered progress in this field (recently reviewed by Hansen and Winter [7]).

**Scheme 1:**
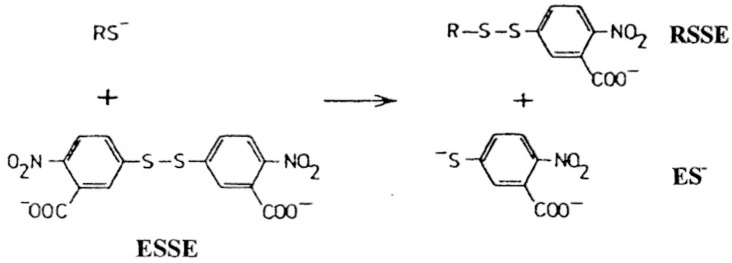
Ellman reagent (ESSE)—reaction with thiols.

In this study, one of the most popular reagents employed for general thiol and glutathione estimation, developed by Ellman [8], utilizing 5’5-dithiobis(2-nitrobenzoic acid) (DTNB or ESSE) has been utilized. This reagent, in conjunction with added enzyme glutathione reductase, has formed the basis of the widely used Teitze assay [9] for the estimation of GSH. ESSE rapidly reacts with thiols in a quantitative fashion at physiological range between pH 7 to 8 as shown in Scheme 1 below.

The yellow anion generated has an extinction coefficient of 14,100 at 412 nm and the mixed aromatic disulfides (RSSE) have extinction coefficients around 8,900 at 325 nm, a property which can be utilized for subsequent analyses [10,11,12]. 

In an attempt to quantify the low molecular weight thiols present in LNCap tumor cells, the mixed aromatic disulfides (RSSE) formed during reaction with the Ellman reagent have been isolated in two steps. This involves selective removal of the RSSE from the acid soluble fraction (ASF) by hydrophobic interaction chromatography followed by ion exchange. The composition of the isolated fractions was checked by capillary electrophoresis analysis. These techniques were used as part of an ongoing program to study the mechanisms by which tumor cells use thiol components to deal with oxidative stress and chemotherapeutic drugs in a human prostate cancer cell line, LNCap clone FGC, obtained from a lymph node metastasis.

## 2. Materials

Unless otherwise stated all chemicals or biochemicals were supplied by Sigma-Aldrich, Poole, Dorset or Merck UK. Only “Analar” or higher grades of purity were used. 4,4’-bis-dimethylaminodiphenylcarbinol (BDC-OH) was supplied by the Nutritional Biochemicals Corp., Cleveland, Ohio. Whatman anion exchange cellulose DE52 was supplied by The Lab Sales Co., Over, Cambridgeshire, UK. and AmberliteXAD-2, a macroreticular, styrene-divinylbenzene copolymer, nonionic bead resin, was obtained from Supelco. ODS-AQ, a C18 reversed phase HPLC packing material based on silica (particle size 50μm), was supplied by YMC EUROPE GMBH.

The ^35^S labeling radio-isotope mixture, Redivue^TM^/Promix^TM^ was obtained from Amersham Pharmacia Biotech, UK Ltd.

### Cell Culture

LNCap clone FGC, ECACC No.89110211 (ATCC CRL 1740), a human, lymph node derived, prostate carcinoma cell line, was purchased from ECACC, Porton Down UK.

Cells were propagated in monolayer culture in RPMI 1640 medium supplemented with 10% fetal bovine serum, 2 mM L-glutamine, 1 mM sodium pyruvate, 10 mM HEPES buffer pH 7.4, 50 μg/mL streptomycin 50 units/mL penicillin in vented tissue culture flasks at 37 °C in a humidified atmosphere of 5% CO_2_.

Cells for analytical work were harvested with the aid of a Falcon cell scraper when approximately 80% confluent. The number of viable cells present was determined by trypan blue exclusion using a Neubauer haemocytometer.

## 3. Methods

### 3.1. Measurement of Total Cellular Thiol Content and Extraction of Acid Soluble Thiols

A suspension was made up of LNCap cells in water (or phosphate buffered saline) at 4 °C to contain 10^7^ to 10^8^ cells per mL using a tight gap (3 thou) all-glass homogenizer. Aliquots of 50 and 100 µL were taken for estimation of total cellular thiol content; for “apparent” thiol content in 100 mM phosphate buffer pH 7.6 containing excess ESSE (100 µg/mL) and for “total” thiol content the cells were dissolved in 8 M urea, 2 M sodium chloride, 0.01 M EDTA, 0.1 M sodium phosphate pH 7.6 also containing an excess of Ellman’s reagent. Suitable blanks made up to allow for any turbidity in the samples or possible A_412_ arising in the ESSE solutions and the OD of the yellow anion (ES^−^) generated was recorded at 412 nm The thiol content calculated using a molar absorption coefficient (ε) of 14,100 M^−1^cm^−1^ (Similar values have been quoted in the literature since the original value of 13,600 M^−1^cm^−1^ given by Ellman but in this laboratory, with the buffers employed, a standard value of 14,100 M^−1^cm^−1^ has been routinely obtained).

For the preparation of the acid soluble fraction an equal volume of 16% w/v trichloracetic acid (TCA) at 4 °C was added and the mixture sonicated for two minutes in ice using a Sanyo MSE Soniprep 150 ultrasonic disintegrator (or until no intact cells could be seen). The homogenate was left at 4 °C about 30 min before centrifuging at 3000 × g at 4 °C until a clear supernatant was obtained (3–4 min). The pellet was re-extracted with half the original volume of 8% w/v TCA and the supernatants combined and filtered to give the acid soluble fraction (ASF). This removed 98% of the “soluble” cellular thiols; a further extraction with 8% TCA removed only an extra 2% of thiol material.

### 3.2. Estimation of the Thiol Content of the ASF

Three different methods were employed each utilizing different chemistries.

(1) Small aliquots, 50 or 100 µL were added to 0.6 M sodium phosphate pH 7.6 (final volume 2 mL) containing an excess of Ellman’s reagent (ESSE) and the concentration of thiol calculated from the yellow anion generated as measured by the absorbance at 412 nm (A_412_)

(2) The thiol content was measured directly on the 8% TCA extract using the Saville method [13], previously used for tumor cell ASF thiols [14], which is based on the formation of S-nitroso derivatives (R-S-NO) from thiols.

The solutions used were: (A) 0.3 g NaNO_2_ in 250 mL water, just before estimation 1 volume mixed with 4 volume of 2 N H_2_SO_4_; (B) 1% w/v ammonium sulfamate; (C) 3.44 g sulfanilamide in 50 mL of 0.4 M HCl and 25 g HgCl in 25 mL 0.4 M HCl, mixed just before use; (D) 0.2% w/v *N*,*N*-naphthyl-ethylenediamine di HCl.

Procedure: To 2 mL 8% TCA extract (*circa* 50–100 nmoles-SH/mL) 0.5 mL A added, then 0.2 mL B mixed/shaken well, 1 mL C followed by 1.3 mL D to give 5 mL final reaction volume. The absorbance was read at 550 nm after 5 min. and the result compared to standard curve obtained from GSH (10–100 nmole aliquots). 

(3) A third method developed by Rohrbach *et al.* [15] was also used. This utilizes another different chemistry at pH 5, the end point being the formation of a thio-ether compound from 4,4’-bis-dimethylaminodiphenylcarbinol (BDC-OH); this can be followed by the decrease in absorption of the parent compound at 610nm (ε = 15,000 M^−1^cm^−1^). 

Briefly, 6.5 mg of BDC-OH were dissolved in 10 mL of AR grade acetone and a 1 to 100 dilution of this stock solution in 50 mM sodium acetate buffer pH 5.1 was used for estimations. 1 mL of thiol containing solution was made up to 10 mL with this reagent and the A_610_ recorded after 30 mins. Standard solutions of thiols such as GSH or sodium 2-mercaptoethane sulfonate were made up to 1.5 µM/mL and a standard curve constructed in the range 0 to 200 µM of thiol. 

The ASF was estimated after removal of TCA with ethyl acetate. For whole cell estimation 4 M guanidinium hydrochloride was included in the pH 5.1 acetate buffer.

The ASF was stored at 4 °C and processed as soon as possible (approximately 30% of the thiol is lost per month of storage at this temperature).

Vicinal dithiols were determined using the basic method of Zahler and Cleland [16] by adding arsenite prior to the Ellman reagent. Dithiothreitol and reduced α-lipoic acid were used as standards.

The disulfide content was determined by modifications of the borohydride method (in borate buffer pH 9.5) followed by estimation of the thiol produced by the Ellman reagent [17].

### 3.3. Preparation and Extraction of Mixed Disulfides for Analysis

The thiol content of the ASF was found to decrease rapidly if the pH of the solution was raised above the 3–4 level attained after removal of the TCA by solvent extraction. To prevent this from happening, the following procedure was devised to minimize the loss of thiols by utilizing their very rapid reaction with Ellman’s reagent to form stable mixed disulfide derivatives suitable for further analysis. All procedures were carried out at room temperature.

The bulk of the TCA was removed by two extractions of equal volume of ethyl acetate and the thiol content of the aqueous layer checked by the methods previously described. From this value the amount of ESSE required to react with the LNCap thiols based on a stoichiometric ratio of approximately 3:1 (excess ESSE) was calculated and this amount of ESSE was dissolved on equal volume 0.6 M sodium phosphate pH 7.8 containing 50 mM EDTA. The ethyl acetate extracted ASF was added to this dropwise with vigorous stirring to give the mixed disulfide (RSSE) and yellow anion ES^−^. The A_412_ and A_325_ (absorption maximum of the yellow anion and aromatic disulfide bond respectively) were checked on completion.

Any residual ethyl acetate was removed by rotary evaporation (or in stream of nitrogen), solid NaCl added until saturated and the mixture immediately applied to a hydrophilic C_18_ reverse phase column of ODS-AQ. 

To prepare standards GSH was dissolved in 8% TCA at approximately the same concentration as the measurable thiol in the ASF and then processed as above. 

### 3.4. Chromatography on ODS-AQ Gel (Reverse Phase C_18_ Silica)

This chromatography was made possible by the development of C_18_ silica packing that can operate in an aqueous environment without organic solvent; ODS-AQ gel. 

Up to 15 mL of the above mixture was immediately applied to a 30 × 2.5 cm column of ODS-AQ gel equilibrated in water at 1 mL/min. Pharmacia HiLoad™ system chromatography equipment was used for the fractionation with a GP-10 gradient programmer and a Uvicord S11 filter with a 313 nm interference filter for monitoring the elution of the disulfides, RSSE. The mixed disulfides were eluted in 200 mL of water followed by a 400 mL linear gradient to 40% methanol. 10 mL fractions were collected. The A_325_ of the fractions was determined using a sipette device on a Cecil 3000 series spectrophotometer.

### 3.5. Chromatography on Anion exchanger—Whatman DE52.

Generally a 30 × 2.0 cm column was found to be satisfactory but care was taken to adequately buffer this material as the Ellman mixed disulfides are unstable above pH 8.5 resulting in the release of the yellow anion (ES^−^)

Pharmacia HiLoad™ system chromatography equipment was also used for this chromatography.

After application of the concentrated pooled fractions from the ODS-AQ column the DE52 was washed with one bed volume of the starting buffer 20 mM Tris-HCl pH 7.2 containing 5% v/v methanol. The aromatic mixed disulfides were eluted in a gradient system employing 20 mM tris-HCl pH 7.2 to 0.35 M guanidinium chloride 20 mM Tris-HCl pH 7.2 containing 5% v/v methanol. 10 mL fractions were collected. Usually 95% + recovery of the A_325_ was obtained.

The A_325_ of the fractions was determined as above and the conductivity measured using a conductivity probe and meter (Hanna HI8820). For convenience the conductivity of the peak fraction with the highest A_325_ was used for identification purposes.

### 3.6 Extraction of ESSE and RSSE from Column Eluates

This was achieved using a specially prepared and optically clean hydrophobic resin, a non-ionic polymeric adsorbent, XAD-2. After removal of methanol and saturation of the DE52 pooled fractions with NaCl, resin was added at 1g per 15 A_325_ units. The mixture was stirred with a suspended stirrer until very little A_325_ (*circa* 5%) was left in the supernatant. When greater than 95% absorption of the RSSE had occurred, as measured by the release of yellow anion from an aliquot of the supernatent after the addition of excess sodium 2-mercaptoethane sulfonate, the resin was filtered off on a sintered glass filter. Usually absorption was complete after 1 to 3 hours. The XAD-2 was then washed with 2 M NaCl until the eluate contained very little UV absorbing material (in the range 200–400 nanometers) and finally with water until the conductivity had dropped significantly and A_325_ started appearing in the eluate.

The adsorbed A_325_ was removed with 50% methanol (20 volumes) overnight to give >90% recovery of the A_325_. The mixture was reduced to low volume 1-2 mL in a rotary evaporator for further analyses.

### 3.7. Capillary Electrophoresis Analysis

A Crystal CE 310 electrophoresis equipment fitted with a Unicam 4225 UV detector (ATI Unicam, Cambridge) and a 65 cm length capillary, ID 75µm was used for these investigations. The temperature of the sample carousel and capillary loop were set and controlled at 25 °C. The most suitable running buffer was found to be 30 mM sodium phosphate pH 7.6 containing 5% isopropanol. 

The capillary pre-wash regimen found to give the best results consisted of: 3 min water, 3 min ethanol:acetone (1:1) and 2 min buffer—all at 2000 mB pressure; a 20 kV pre-run in buffer for 2 min. The sample was applied in 0.2 min at 20 mB pressure. After sample electrophoresis at 20 kV for up to 40 min, the capillary was washed with 0.5 M sodium hydroxide for 1 min followed by water for 1 min (all at 2000 mB).

Each sample run, consisting of up to eight samples, contained at least two runs of a mixed standard containing ESSE and GSSE (*circa* 5–10 A_325_ units/mL).

### 3.8. ^35^S Labeling of Cellular Thiols

Cells for short-term ^35^S labeling studies were propagated in monolayers, usually in T175 flasks and used for labeling studies when about 80% confluent. 

In order to deplete intracellular pools of methionine, after draining off the growing medium, the cells were incubated for 20 min in a similar RPMI medium (Sigma R7573) which was deficient in methionine and cysteine. It contained 5% FBS, 2 mM L-glutamine, 1 mM sodium pyruvate, 10 mM HEPES buffer pH 7.4. 

After draining off the depletion medium a similar volume was added containing the Redivue^TM^ (Promix^TM^) ^35^S *in vitro* cell labeling mixture (Amersham Pharmacia Biotech UK Ltd., specific activity 800–1200 Ci/mmole) which contains ^35^S methionine and ^35^S cysteine in a ratio of approximately 70:30. The optimum level of isotope for a 4 hour label was found to be in the region of 250 μCi per T175 containing just over 10^7^ cells. The flasks were incubated at 37 °C for 3 hours with occasional tilting, then opened in a designated fume hood equipped with an activated charcoal filter. After a wash with a small quantity of cold deficient medium the flasks were drained as completely as possible and the cells from each flask were scraped off into 2 mL of cold PBS. Aliquots taken for ^35^S and total thiol determination before preparing the ASF, *etc.,* as described above.

For determination of ^35^S counts, up to 500 μL of sample, dissolved a suitable aqueous solvent, were mixed with 4 mL of Packard LSC Ultima Gold liquid scintillation cocktail mixture to give a homogeneous clear solution before counting in a Packard Tri-Carb 2100TR liquid scintillation analyzer to give corrected dpm values.

## 4. Results and Discussion

*4.1 General Thiol Content*

The thiol content of these cells and the ASF obtained is given in Table 1 below.

**Table 1:** LNCap cellular thiol content—femtomoles per cell.

Whole Cells (Ellman method)	“Apparent” 17.3 ± 3.1 (5)
	Total 35.1 ± 2.2 (7)
Acid soluble fraction (ASF) (8% TCA extract)	Ellman (ES-) 5.3 ± 0.5 (8)
	Saville (RS-NO) 5.1 ± 0.6 (7)
	Rohrbach (RS-C-BDC) 5.5 ± 0.3 (4)
	Average value 5.3

Values given as means (±SEM) of the number of determinations given in parentheses.

The ASF thiol values, estimated by completely different chemistries, confirmed that the thiol content of the ASF as measured by the Ellman reagent was a true value and not as a result of the presence of interfering substances or non-specific interactions with the reagents employed. For example, the Ellman reagent will react with reducing sulfur containing anions such as sulfide and bisulfite, whereas the Saville assay does not record a colored end-point with these anions. 

(Also, the absence of nitroso-thiols in the ASF could be demonstrated by adding Saville reagent solutions C and D directly to the ASF—no magenta dye was formed). Further confirmation of the specificity of these reagents with this ASF was provided by the Rohrbach reagent that uses the colored/magenta BDC-OH reagent to form colorless thioethers with thiols. 

Further investigation of the ASF thiols revealed the following important information:

(a) Addition of sodium arsenite to vicinal dithiols such as DTT or reduced α-lipoic acid prior to the Ellman reagent delays the formation of yellow anion by the formation of a transient complex of cyclic dithioarsenite [16]. However, arsenite addition to the LNCap ASF prior to addition of ESSE had no effect on the A_412_ generated from this extract, demonstrating the absence of vicinal dithiols in the mixture. (By contrast the total cell thiol was reduced initially by some 30–40% by adding arsenite prior to the Ellman reagent to whole cells dissolved in 6 M urea 2 M NaCl.). This is a significant finding as the presence of vicinal dithiols in the ASF would affect the formation of mixed disulfides with ESSE since the first formed aromatic mixed disulfide, being in a local concentration of 6–10 M with the second thiol will favour the liberation of the second molecule of ES^−^ [18]. This would result in the formation of an internal disulfide and not the RSSE expected. 

(b) Many investigators have reported the presence of low levels of disulfide in the ASF of various cells, sometimes as a result of post extraction oxidation. Reduction of the LNCap ASF with the strong reducing agent sodium borohydride resulted in very little increase in thiol value indicating that practically no disulfide is present in this extract. (This reagent effectively gave 100% reduction of α-lipoic acid at pH 9–10). This result serves to confirm that TCA is one of the most suitable and convenient non oxidizing reagent for protein denaturation [7].

(c) Progressive loss of thiol occurs as the pH is raised after removal of the TCA with ethyl acetate. 45% ± 5% of the thiol is lost after raising the pH to 7–8 and this rises to 90%+ at pH 9–9.5. Reduction of the later solution with NaBH_4_ only restores 31 ± 3% of the original thiol value indicating that the bulk of the -SH was not converted to disulfide which is the usual product of the mild oxidation of thiols.

In these studies, the ASF thiol was always added to a three-fold excess of ESSE so it is difficult to envisage how these residual thiols could be converted to disulfides; particularly as no vicinal dithiols could be detected in the ASF. In the absence of any free thiol in the reaction mixture at any time, disulfide exchange will not normally occur. 

### 4.2. Examination of Products of Reaction with Ellman Reagent

An effective method for the bulk isolation of the components of the reaction of thiols with ESSE was evolved from earlier work on hydrophobic interaction chromatography of the mixed aromatic disulfides RSSE and ESSE. In these studies C_18_ reverse phase hydrophilic silica, ODS-AQ was effectively utilized for this purpose on the mixture as soon as possible after carrying out the reaction. In this system the yellow anion ES^-^ passes straight through the column together with the salts and other low molecular weight components such as nucleotides. This step was found to be essential as, at the initial stages of this work, as it was found that, in time, the nucleophilic character of the ES^−^ allows it to attack the free carbons of the benzene ring of the unreacted ESSE. This yielded disulfide artefacts giving multiple bands on capillary electrophoresis analysis. Compounds containing molecular ions of 592 m/z (ESSE + ES^−^) and 789 m/z (ESSE + 2ES^−^) were isolated from ESSE / ES^−^ mixtures. The formation of these compounds is probably the main reason that the yellow anion often fades rapidly in ESSE/thiol reaction mixtures rather than the oxidation of the ES^−^ ion postulated by some authors [12] 

**Figure 1:**
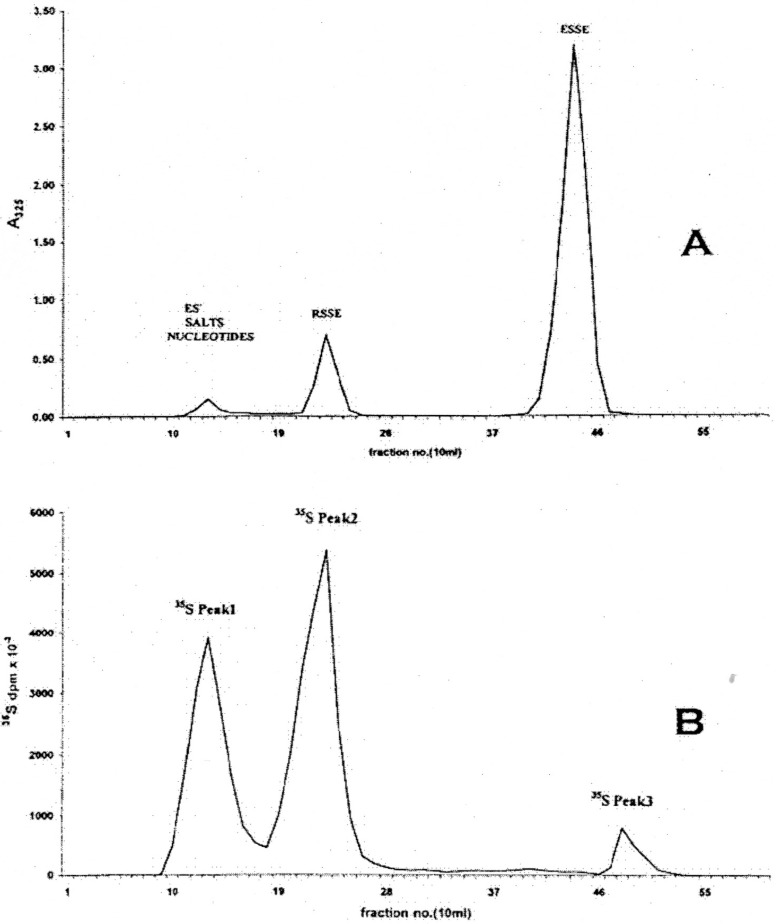
Initial chromatographic separation of LNCap cell acid soluble thiol reactants with a three-fold excess of ESSE on a 30 × 2.5 cm column of ODS-AQ gel using 10–15 mL of sample saturated with NaCl. Elution: flow rate 1 mL/min, 200 mL water followed by a 400 mL linear gradient to 40% methanol; 10 mL fractions. **(A)** 325 nm absorbency trace from a bulk preparation; **(B)** ^35^S trace from cells labeled in monolayer (*circa* 80% confluent) for 4 hours.

The separation of the reaction components on an ODS AQ silica gel column is illustrated in Figure 1. 

**Figure 2:**
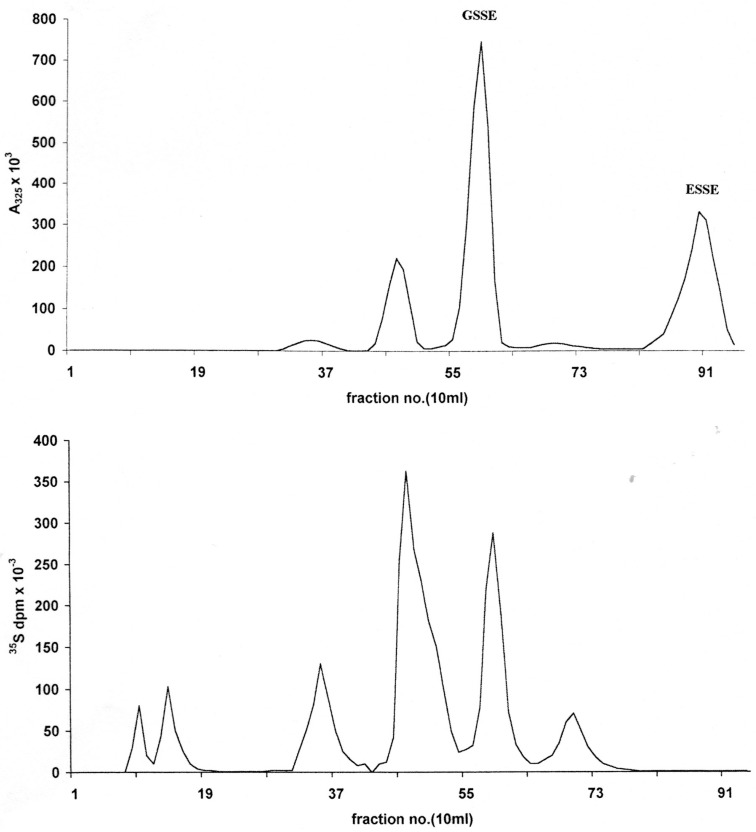
Anion exchange chromatography of RSSE isolated from LNCap ASF on ODS-AQ (peak 2) using a 30 × 2 cm column of DE52 cellulose. Elution consisted of a 125 mL wash with 20 mM Tris-HCl pH 7.2 containing 5% v/v methanol followed by an 800 mL linear gradient to 0.35 M guanidinium chloride 20 mM Tris-HCl pH 7.2 containing 5% v/v methanol at a flow rate of 1 mL/min. 10 mL fractions were collected. Upper trace—Applied 60 A_325_ units of RSSE: abscissa A_325_ × 10^3^; Lower trace—15 A_325_ units/3.5 × 10^7^ dpm R35SSE: abscissa 35S dpm × 10^3^.

**Figure 3:**
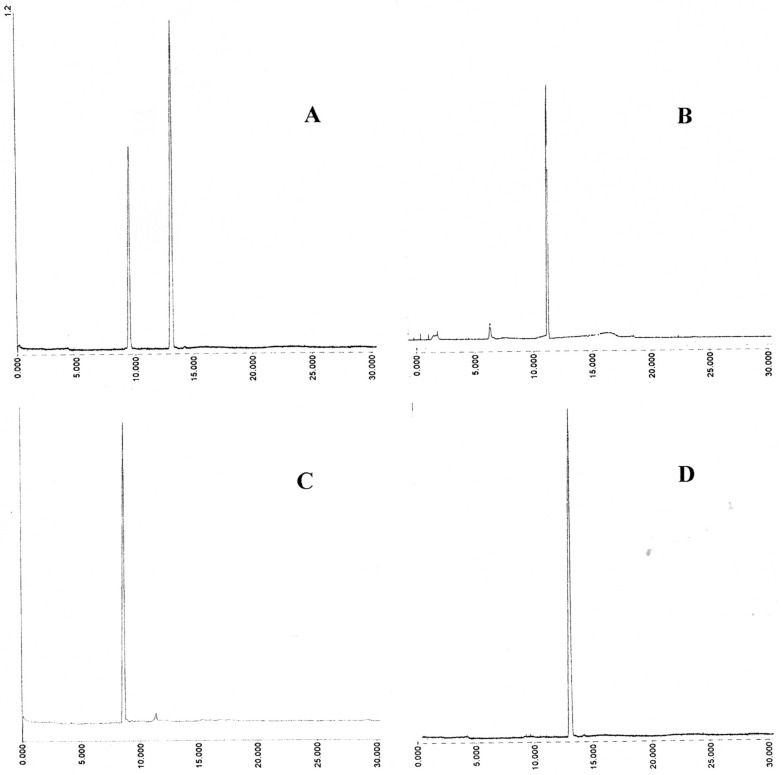
Capillary electrophoresis of aromatic mixed disulfides isolated on DE52. Instrument: Crystal CE310 (ATI Unicam) capillary; length 65 cm, ID 75 μm; UV detector; Unicam 4225 set at 325 nm; Buffer; 30 mM sodium phosphate pH 7.6 containing 5% isopropanol; Applied voltage 20 kV; Temperature 25 °C; Injection of sample; 0.2 min at 20 mB pressure. **(A)** GSSE (left) and ESSE (right); **(B)** 12.5 mS peak; **(C)** 15.1 mS peak; **(D)** 25.2 mS peak.

After an initial elution with water, application of a water-methanol gradient resulted in the quantitative elution of the RSSE followed by the excess/unused ESSE (Figure 1A). The identity of the latter as the sole component of this peak was confirmed by DE52 chromatography (Figure 2) and capillary electrophoresis (Figure 3D). 

However, under the low salt conditions employed, some ESSE was also found to be bound to the RSSE fraction as shown by re-chromatography of this on DE52 anion exchanger (Figure 2, top trace). In addition to this excess ESSE, eluted at the conductivity of 25.2 mS, two major and two minor RSSE components were detected. As expected, the largest component was GSSE, which elutes at 15.1 mS. 

Examination of the first unabsorbed ODS-AQ peak (Peak 1) (Figure 1) indicates that the bulk of the thiol seems to have been converted into another form, not the RSSE/mixed aromatic disulfide expectedas this peak had virtually no UV absorbency at 325 nm. However, the presence of this sulfur containing reaction product could be demonstrated by the 4-hour ^35^S labeling pattern obtained on ODS-AQ (Figure 1B). Although this peak constituted 42.7% of the ^35^S present in the ASF it perhaps represents >60% of the actual ASF thiol, since, by a summary of the peaks detected by DE52 chromatography (Figure 2 upper trace), only approximately 40% of the measured RSH can be accounted for as RSSE. 

Gel filtration of peak 1 on a Biogel P2 column, which has a fractionation range of 100 to 1800 daltons, revealed the presence of at least three major and three minor ^35^S peaks. Further examination of these peaks may throw some light on the nature of these sulfur compounds or reaction products.

### 4.3. Calculation of Glutathione Content

To check that GSH could be quantitatively recovered in this analysis system, quantities of GSH equivalent to those found in the ASF, were dissolved in 8%TCA and put through the processing described. As a result of four runs GSSE recoveries were found to be in excess of 85% based on the amount of yellow anion released by 2-mercaptoethane sulfonate. From the ratio of A_325_ to thiol value (calculated from the A_412_ released) the molar extinction coefficient at 325 nm was found to be 8,990 ± 210 M^−1^cm^−1^

The pattern obtained from DE52 anion exchange chromatography of the RSSE isolated on ODS-AQ as peak 2 (Figure 1) is shown in the upper trace of Figure 2. From the A_325_ recovered it was calculated that the GSSE peak (15.1 mS) could only account for about 28.5% of the total thiol present in the ASF (see Table 2) or 4.5% of the total cell thiol. 

Apart from the major GSSE peak and the non-specifically bound excess ESSE (at 25.2 mS) there are three other minor components at 9.5, 12.5 and 17.2 mS.

Capillary electrophoresis was carried out where sufficient material could be isolated after extraction of the pooled peaks with XAD-2. Russell and Rabenstein [19] have also reported the CE analysis of RSSE derived from standards and natural sources/erythrocytes) but without the advantages of the extraction procedures developed here.

In the CE system employed the relative mobilities (R_M_) of a set of prepared standards, as measured by the time of emergence divided by the time of emergence of ESSE, were as follows: CysteamineSSE 0.38 ± 0.01 (6), CysteineSSE 0.67 ± 0.01, glutathioneSSE 0.74 ± 0.02 (ESSE 1.00), coenzymeA-SSE 1.18 ± 0.04 (all results were the mean of six to eight runs). The CE traces for the LNCap RSSE are given in Figure 3; by comparision with prepared standards the 15.1 mS peak (C) had an (R_M_) of 0.73 ± 0.03 confirming it as GSSE and the 25.2 mS (D) of 1.00 ± 0.02 as ESSE . The 12.5 mS peak seemed to consist largely of one component which had a relative mobility against ESSE of 0.84 ± 0.06. 

### 4.4. ^35^S Labeling Studies

Table 2 summarizes the data obtained from the ^35^S labeling of LNCap cells. 

It is notable that on DE52 analysis of the labeled mixed disulfides only 27.2% of ^35^S was found to be incorporated was into G^35^S SE, and this represents only 9.9% of the total S present in the ASF, indicating that GSH is not the most metabolically active thiol in this cell line. This, coupled with the finding that the GSSE was found to account for 28.5% of the total ASF thiol, would seem to indicate that GSH is not the major non-protein thiol of these tumor cells. 

**Table 2:** Glutathione content and ^35^S labeling in LNCap cells using the Ellman Reagent

GSH content of cells by DE52/15.1mS analysis of RSSE	(a) Whole cells 1.5 ± 0.2 femtomoles/cell (b) GSH content as percentage of ASF thiols 28.5 ± 4.3% (c) GSH content as percentage total cell thiol 4.5 ± 0.4%	
^35^S Promix (Redivue) cell labeling of cells 4 hours at 37 °C	(a) Percentage of ^35^S label taken up by cells (b) Percentage of incorporated ^35^S present in ASF (c) ^35^S dpm × 10^−8^/μmole–SH in ASF (d) ^35^S dpm × 10^−8^/μmole –SH in whole cells	18.8 ± 2.2% 9.9 ± 0.9% 3.1 ± 0.8 8.0 ± 0.6
Chromatographic Analysis of R^35^SSE	(a) Percentage ^35^S **adsorbed** on ODS-AQ (=R^35^SSE) (b) Percentage R^35^SSE in form of G^35^S SE = DE52/15.1mS peak (c) Percentage of total R^35^SSE in form of DE52/12.5mS peak (d) Percentage of **total ASF–^35^S** in form of G^35^S SE	54.7 ± 2.2% 27.2 ± 2.5% 46.5 ±12.2% 8.4%

Results given as means (±SEM) of 5-6 experiments.

The trace DE52 peaks at 9.5 and 17.2 mS were present in too small a quantity to permit CE analysis in this study; however the ^35^S pattern (lower trace Figure 2) confirmed their presence together with two further peaks (*circa* 6 and 7 mS) virtually unadsorbed on DE52. These are obviously trace thiols of high metabolic activity, the presence of which has been reported in a previous study [20]; in which E^35^S-^35^SE labeled reagent, was used to label rat liver nuclear thiols to give RS-^35^SE mixed disulfides. 

Of all the R^35^S-SE components isolated on DE52, the 12.5mS fraction has the highest specific activity (most heavily labeled), but part of this could be ^35^S cysteine (Cysteine^35^SSE that elutes at 12.0 mS) as this was originally present in the Redivue labeling mix. It should be noted that no ^35^S was detected in the 25.2 mS area where ESSE emerges. 

The state of the art in the determination of glutathione and its disulfide in biological samples and the difficulties involved has been recently reviewed [21] With regard to GSH levels of cultured tumor cells considerable variations have been reported depending on the stage of cell growth/proliferation to the method of GSH estimation employed. Eady *et al*. [22] for example, reported large fluctuations on a daily basis (0 to 30 femtomoles/cell) in six human tumor lines. El-akawi *et al.* [23] reported levels of tumor GSH which correlated with resistance to chemotherapeutic drugs; 3 femtomoles/cell in original tumor, 9–11 femtomoles/cell in resistant lines. Similar results have been reported by other groups: Batist *et al.* [24] found 2.4–5.9 femtomoles/original tumor cell rising to 7.5–24.2 femtomoles/resistant tumor cell; Xu and Thornalley [25] reported values of GSH in human leukaemia p53- and p53+ cells as 1.94 and 2.5 femtomoles/cell, respectively. 

## 5. Conclusion

The studies reported here show that, although the Ellman reagent releases quantitative amounts of ES^−^ which are representative of the thiol present in the ASF, an equivalent amount of mixed disulfide is not formed. Very similar results to those reported above have been obtained with mouse Ehrlich ascites tumor cells where GSH constituted 42 ± 3% of the total cellular thiol (unpublished data). It appears that the bulk of the low molecular weight thiol present in this tumor is unable to form a mixed disulfide or that, on formation, it is so unstable that it rapidly breaks down, releasing the sulfur fragment (s) as seen in the ^35^S study reported here. The identification of these unknown highly reactive thiols poses an exciting challenge for the future. If the findings reported in this paper can be confirmed by other analytical techniques, we may be on the verge of obtaining a new and important tool to provide an insight into the metabolic control of tumor cells, perhaps revealing some fresh, long-hoped for weakness in tumor cell metabolism that can be utilized for radio- and chemotherapy treatments.

## Abbreviations

ASF: acid soluble fraction obtained after deproteinization of cells;
DTNB or ESSE: 5’5-dithiobis(2-nitrobenzoic acid);
RSSE: aromatic mixed disulfide (s) formed on reaction of cellular thiols with Ellman’s reagent;
ES: yellow anion, 5-thio-2-nitrobenzoic acid;
GSH: glutathione;
mS: milli-Siemens conductivity;
TCA: trichloracetic acid.
